# Medical and Physical Intelligence

**Published:** 1823-04

**Authors:** 


### MEDICAL AND PHYSICAL INTELLIGENCE.

### PHYSIOLOGY.

1. *Communication between the Stomach and Bladder.*—The experiments made in England by Darwin, and more recently by Wollaston, Brande, and Marcet, tended to prove that different substances introduced into the stomach were found in the urine, without having passed by the medium of the lymphatics or blood-vessels. M. Fodéra has resumed these experiments; and he has given to them an ingenious modification, which enabled him to discover some phenomena which had escaped the English physiologists. He introduced into the bladder a catheter, with a cork, having tied the penis that the urine might not flow by the sides of the instrument. He laid bare the œsophagus at the anterior part of the neck, and injected into the stomach a solution containing some grains of the hydrocyanate of potass, with iron. This being done, he frequently uncorked the sound, and received the urine which flowed from it on paper. He placed a drop of solution of sulphate of iron on the paper, and added to it another of hydrochloric acid, to make the colour disappear. In one experiment, the prussiate was detected in the urine ten minutes after its injection into the stomach; and, in another, five minutes thereafter. The animals were instantly opened. The salt was found in the serum of the blood drawn from the thoracic portion of the inferior vena cava; in the right and left cavities of the heart, in the aorta, the thoracic duct, the mesenteric ganglions, the kidneys, the joints, and the mucous membrane of the bronchiæ.—(*Journal de Physiologie*, Janvier, 1823.)

2. *Transudation.*—The following experiments prove that, in the dead body at least, transudation of fluids may take place at the same time from within outwards, and from without inwards, through the parietes of the intestines and of blood-vessels. M. Fodéra filled a portion of the intestine of a rabbit with a solution of prussiate of potass, and plunged it into a solution of the hydrochlorate of lime: he introduced into it another portion of hydrochloric acid, and surrounded it with sulphuric acid; finally, he placed a bladder filled with fluid, coloured with turnsol, in a solution of galls. Some time after he found in the interior of these, respectively, hydrochlorate of lime, sulphuric acid, and gallic acid, recognized by the nitrate of silver, hydrochlorate of barytes, and sulphate of iron; and, in the liquids in which he had immersed them, prussiate of potass, hydrochloric acid, and turnsol, recognized by the sulphate of copper, nitrate of silver, and by the colour of the infusion of galls being rendered bluish by the potass.

Similar phenomena were witnessed on the living body; and the rapidity of the transudation was found to be much increased by the application of galvanism. For this purpose, the ingenious experimentalist injected into the bladder, or into a portion of intestine, in a living rabbit, a solution of the prussiate of potass, which was made to communicate with a copper wire: he placed on the exterior surface a piece of linen, moistened with a solution of the sulphate of iron, which communicated with an iron wire: these wires were brought into contact with those of the battery. If the galvanic current be directed from without inwards, by making the iron wire communicate with the positive pole, and the copper with the negative, the tissues of the organs become imbibed with Prussian blue; but, if the current be changed, the colour is manifested on the linen.—(*Journal de Physiologie*, Janvier 1823.)

---

### PATHOLOGY.

3. *Bones of a Fœtus voided by the Rectum.*—Catherine Damiano, of healthy constitution, after two favourable accouchements, became pregnant for the third time. This conception was marked by all the usual symptoms, the enlargement being inclined towards the left iliac region. The existence of the fœtus was demonstrated by its movements: these were sometimes interrupted, and often very sensible in this region. At this time she was taken with pains, which were believed to be those of labour, and which ended in an evacuation from the uterus, of a liquid slightly tinged with blood. The milk-fever supervened; the mammæ were filled, and the secretion flowed from them in abundance. These occurrences took place in November; and, from December till August following, she menstruated regularly: at this period the evacuation ceased, without any apparent cause. She had a continual discharge from the vagina, of a whitish-yellow fluid, and experienced various inconveniences. She continued to be tormented by very acute pain, corresponding to the anterior part of the tumor and the eminence of the sacrum, and by continual diarrhœa, with tenesmus. Towards the middle of January of the second year, she suffered more than ever, and was seized with continued fever, with intervals of cold followed by burning heat, which was felt particularly towards the sacrum: she then evacuated by the rectum some bones, naked, deprived of cartilage, and without any sensible figure. The excretion of these bones was accompanied by that of some purulent sanguineous matters. In examining the parts carefully, M. Roagna discovered that these bones had made a route for themselves, by means of an opening in the rectum, about six lines in length, and about twenty-one lines from the anus. This evacuation greatly relieved the patient; but the pains returned with violence, and at several times, until all the bones were voided, which took place in the month of July, the third year after the conception.—(*Revue Medicale*, Janvier 1823.)

4. *Fœtus affected with dry Gangrene of the lower Extremities.*—Madame N., after having borne several children, and several times miscarried, was brought to bed, in the sixth month of her pregnancy, of a male infant, living and externally well formed, and healthy from the hypogastric region upwards, but having the parts of body from this region downwards affected with dry gangrene. It lived two hours. As the body was not opened, the cause of this phenomenon was not ascertained, but it is conjectured to have arisen from malformation of the internal iliac arteries preventing the circulation in the inferior parts, which fell into a state of gangrene in consequence of this want of nourishment.—(*Ibid.*)

### THERAPEUTICS.

5. *Cramp in the Chest.*—A case of cramp in the chest (angina pectoris?) which had withstood all other remedies, was cured by the use of the Extractum Lactucæ viros. After this it was frequently employed in similar cases, and always with like benefit.—(*Petersburger Vermischte Abhandlungen.*)

6. *Perspiration of the Feet.*—When the perspiration of the feet is checked, and unpleasant symptoms are thereby produced, the natives of Finland are in the habit of restoring it by means of the exterior white bark of the beech (*betula alba*). It is worn in the form of soles to the shoes, the inner surface of the bark being turned to the planta pedis.—(*Ibid.*)

---

### SURGERY.

7. *Chronic Ulcers.*—La Charité, the principal hospital at Berlin, has long been celebrated for the cure of chronic ulcers of the legs, and the method adopted seems chiefly to consist in restricted diet. The plan is as follows:—On admission into the hospital, the patient is purged, and put into a bath; he is then placed in a ward assigned for such cases, and confined to very spare diet, while cold applications are made use of to the sore. The bath, and a purgative of calomel and jalap, are repeated twice a-week. By this treatment the ulcers soon acquire a better appearance, and, even if very foul and extensive, they generally heal in a month or six weeks. Great attention is paid to the state of the pulse, which is examined every day, because it affords an indication by which to judge whether the treatment is to be carried on or not: if it be reduced to 35 or 40, the starving system is discontinued, and somewhat better diet allowed, until the pulse becomes natural again; as, otherwise, blindness, ringing of the ears, giddiness, fainting, &c. are liable to be produced. If attention is paid to this, no other danger is to be apprehended in the progress of the cure. *Another advantage of this plan is, that it is exceedingly cheap.*—(Rust's *Mag. der Heilkunde,* 9 b.)

8. *Means of Breaking down Calculi in the Bladder.*—An instrument has been invented, and it is said brought to perfection, in Paris, by M. Amusat, the use of which is to break down calculi in the bladder, and render the fragments so small that they may be voided as gravel. The instrument consists of pincers, which are confined in a tube not larger than a sound, until introduced into the bladder. They are then opened, the stone is seized with facility, and, by moving the handles in a particular manner, is soon reduced to powder. In a few seconds, a stone the size of a nut is broken with facility. It appears, however, that as yet the trial has been made only on a *dead* body: it still remains to be learned what the result will be in a living one.—(*Journal of Science.*)

---

### PHARMACY.

9. *Hydrocyanic Acid.*—Some chemists, as Lampaduc and Brugnatelli, formerly announced that the hydrocyanic acid might

3

be obtained from prussiate of potass; but they did not sufficiently determine the manner in which it might be rendered of uniform strength. M. Gea Pessena, a chemist at Milan, has employed himself in removing this deficiency. The following is his process, which must be economical, if the result corresponds with the account of the inventor. He introduces eighteen parts of prussiate of potass and iron, reduced to a very fine powder, into a small tubulated glass retort, taking care not to soil the neck or side. He adapts to this vessel a very small tubulated balloon, furnished with a conducting tube, which he plunges into the first flash, containing a little distilled water. The rest of the apparatus is contrived so as to avoid absorption. This being done, he pours into the retort a cold mixture, of nine parts of concentrated sulphuric acid and twelve parts of water. The tube of the retort is to be hermetically secured; the whole left at rest for twelve hours, at the commencement of which the balloon is to be surrounded with ice; the neck of the retort is to be constantly cooled with wet cloths; afterwards the materials are to be heated with some burning charcoal, and preserved in this state until the streaks, which are observed in the neck of the retort, become more rare, and until a blue vapour arises, which threatens to pass into the receiver. At this point, the heat is immediately discontinued; the apparatus is allowed to cool entirely, and the contents of the receiver are poured into a proper vessel. The hydrocyanic acid obtained by this process has a strong and penetrating odour; the specific gravity is from 0.898 to 0.900, at the temperature of 13 or 14 of Reaumur; it likewise possesses, according to M. Pessena, all the properties of the purest prussic acid.—(*Giornale de Fisica*, 1822.)

10. *Hydriodate of Potass.*—The importance of preparing the various salts of iodine used in medicine with accuracy, is too obvious to require comment; we therefore regard the following directions respecting the hydriodate of potass as meriting attention:

One part of iodine, and three or four parts of water, are to be introduced into a phial or matrass; by degrees, and at intervals, an excess (one-half part, for example,) of pure filings of iron is to be added. The combination immediately takes place; much heat is disengaged; the iodine disappears, and the liquid becomes of a deep red colour. During this violent re-action, a hydriodate, with excess of iodine, is formed, which has only to be heated, and constantly and slightly agitated, to be converted into a simple hydriodate of iron. The action is shown to be terminated by the almost complete absence of colour in the liquid, but still more certainly by white paper being no longer stained red by it. The liquid is to be filtered, and to be augmented by some parts of water; it is to be placed in a sand-bath, in a retort or matrass, and nearly at the boiling point; then the iron is precipitated by means of pure carbonate or subcarbonate of potass. This part of the process requires some attention, lest an excess of potass be added; which, however, may be separated by repeated crystallizations, or saturated with hydriodic acid. After having filtered, in order to separate the ferruginous precipitate, and having washed it well, it is to be evaporated. The salt may be made to crystallize by cooling or by evaporation: in this latter case, the concentrated solution of the hydriodate of potass is to be placed, not upon a stove, because the salt would form along the sides of the vessel, and the liquid in the end be entirely remixed,—but on a very gentle fire, where the edges of the vessel, being less heated than the bottom, may condense a little of the vapour which rises, and thus prevent the ascension of the salt. By degrees, crystals are deposited; when they fill almost all the space occupied by the liquid, it must be allowed to cool; then the mother water is to be poured off, and again evaporated to obtain more of the salt; finally, the crystals are to be entirely dried at a stove or fire, where they undergo a slight decrepitation.—(*Journal de Pharmacie, &c.* Janvier 1823.)

11. *Castor Oil.*—The processes generally employed for the extraction of the oil from the seeds of the ricinus, are by boiling in water and by expression. The former is expensive, from the quantity of fuel it requires, and affords but a small proportion of oil, which becomes in some degree empyreumatic, from the heat necessary to separate the mucilage. By the second, a milder oil is obtained, it is true, but it is extracted with difficulty, from its viscidity and the great quantity of mucilage which accompanies it. These difficulties are overcome by the following process, founded on the property possessed by alcohol of dissolving the oil, and separating the mucilage. It consists in mixing the seeds, deprived of their rind, and beat into a paste with a certain quantity of alcohol, (four ounces to the pound,) at 36° (of the centigrade thermometer); this mixture is subjected to the press; the liquid flows with great facility, and is afterwards distilled; the residue of the distillation washed with many waters. The oil separated by the water is placed upon a gentle fire, to evaporate all the moisture; it is then taken from the fire, and thrown upon filters placed upon a stove heated to 30°; it filters freely, and the oil obtained is very clear and very mild. The products constantly obtained in this way, by M. Faguor, are ten ounces of oil from each pound of the peeled seeds, and only seven ounces with the rinds on. These products greatly exceed the quantity obtained by the old methods. M. Henry obtained a still larger proportion of oil, the difference probably depending on the superior force of his press. Half the quantity of alcohol employed may be recovered by the distillation.—(*Journal de Pharmacie,* October 1822.)

12. *On a new Acid produced by the Distillation of Citric Acid,* by J. L. Lassaigne.—When citric acid is put to distil in a retort, it begins at first by melting; the water of crystallization separates almost entirely from it by a continuance of the fusion, then it assumes a yellowish tint, which gradually deepens. At the same time there is disengaged a white vapour, which goes over to be condensed in the receiver. Towards the end of the calcination a brownish vapour is seen to form, and there remains at the bottom of the retort a light, very brilliant charcoal.

The product contained in the receiver consists of two different liquids. One, of an amber-yellow colour, and an oily aspect, occupies the lower part; another, colourless and liquid like water, of a very decided acid taste, floats above. After separating them from one another, we perceive that the first has a very strong bituminous odour, and an acid and acrid taste; that it reddens powerfully the tincture of litmus; but that it may be deprived almost entirely of that acidity by agitation with water, in which it divides itself into globules, which soon fall to the bottom of the vessel, and are not long in uniting into one mass, in the manner of oils heavier than water.

In this state it possesses some of the properties of these substances; it is soluble in alcohol, ether, and the caustic alkalis. However, it does not long continue thus: it becomes acid, and sometimes even it is observed to deposit, at the end of some days, white crystals, which have a very strong acidity. If we then agitate it anew with water, it dissolves in a great measure, and abandons a yellow or brownish pitchy matter, of a very obvious empyreumatic smell, and which has much analogy with the oil obtained in the distillation of other vegetable matters. The same effect takes place when we keep it under water; it diminishes gradually in volume, the water acquires a sour taste, and a thick oil remains at the bottom of the vessel.

This liquid may be regarded as a combination (of little permanence, indeed,) of the peculiar acid which the oil formed in similar circumstances.

As to the liquid and colourless portion which floated over this oil, it was ascertained to contain no citric acid *carried over*, nor acetic acid: first, on saturating it with carbonate of lime, a soluble calcareous salt was obtained; and, secondly, because this salt, treated with sulphuric acid, evolved no odour of acetic acid.

From this calcareous salt the lime was separated by oxalic acid; or the salt itself was decomposed with acetate of lead, and the precipitate treated with sulphuretted hydrogen. By these two processes, this new acid was separated in a state of purity.—*(Journal of Science.)*

---

### MISCELLANEOUS.

13. *New Faculty of Medicine of Paris.*—The King of France, by an ordonnance just published, has re-organized the Faculty of Medicine of the Academy of Paris. This official document is of great length; but we have extracted the principal facts contained in it, which may be interesting and useful to our readers to be acquainted with. The Faculty of Medicine is to be composed of twenty-three professors; thirty-six associates *(agregés)* are attached to these, two-thirds of whom are in exercise; and there is also an indeterminate number of free associates. The rank of associate can only be conferred on doctors in medicine or surgery who have reached the age of twenty-five. The professors on this occasion are appointed by the king, and the two-thirds of the associates by the grand master of the university. In future, when there is a vacancy for the place of professor, three candidates are to be presented by the assembly of the Faculty, three by the Academic Council, (both taken from among the associates,) and the nomination shall take place from among these by the grand master, according to the regulations of the university. The professors and associates of the other Faculties of Medicine in the kingdom may also be named as candidates.

The chairs in the Faculty of Medicine are the following:—1, Anatomy; 2, Physiology; 3, Medical Chemistry; 4, Medicine; 5, Natural History (medical); 6, Pharmacology; 7, Hygiene; 8, Surgical Pathology; 9, Medical Pathology; 10, Operations and Dressings; 11, Therapeutics and Materia Medica; 12, Legal Medicine; 13, Midwifery and Diseases of Women and Children. Two professors are attached to the chair of surgical pathology, two to that of medical pathology, and one to each of the others.

Relative to the admission of pupils, the following regulations are to be observed:—No ticket will be granted unless, 1st, the register of birth is produced; 2d, a certificate of good conduct and morals from the mayor of the commune, and confirmed by the prefect; 3d, the diploma of bachelor in letters and sciences; and, 4thly, if a minor, the consent of his parents or guardians.

The second ordonnance gives the names of the professors attached to the different chairs of the Faculty, as follow:—Messrs. Beclard, anatomy; Dumeril, physiology; Orfila, medical chemistry; Peletan, jun. medicine; Clairon, medical natural history; Guilbert, pharmacology; Bertin, hygiene; Marjolin and Roux, surgical pathology; Fizeau, medical pathology; Richerand, operations, &c.; Alibert, therapeutics and materia medica; Royer Collard, legal medicine; Desormeaux, midwifery and diseases of women and children; Recamier, Laennec, Landré, Beauvais, and Cayol, medical clinics; Boyer, Dupuytren, and Boujon, surgical clinics; Deneux, midwifery clinics.

Messrs. De Jussieu, Vauquelin, Dubois, Pelletan, sen. Deyeux, Pinel, Desgenettes, Chaussier, Lallemand, Le Roux, and Moreau, are named honorary professors. The associates (on exercise) appointed by the grand master of the university of Paris, in conformity with the above decree, are Messrs. Adelon, Alard, Arvers, Breschet, Capuron, Chomel, Cloquet (Hyppolite), Coutanceau, De Lens, Gaultier de Claubry (Emmanuel), Guersent, Jadioux, Kergaradec, Maisonabe, Moreau (F. Joseph), Murat, Parent du Chatelet, Pavet de Courteille, Rateau, Richard (Achille), Rullier, Segalas, Serres, Thevenot du Saint Blaise. By another arrêt of the grand master, M. Landré Beauvais is named dean of the Faculty.

14. *Prize Essay.*—The subject of the essay, proposed by the Medical Society of London, for the Fothergillian gold prize of the ensuing year, is "Diseases of the Spine."

*Conditions.*—The Society resolve to give annually to the author of the best dissertation on a subject proposed to them, a gold medal, value twenty guineas, called the Fothergillian medal, for which the learned of all countries are invited as candidates. 2. Each dissertation offered for this prize must be delivered to the registrar, in the Latin or English language, on or before the 31st day of December. 3. With it must be delivered a sealed packet, with some motto or device on the outside, and within the author's name and designation; and the same motto or device must be put on the dissertation, that the Society may know how to address the successful candidate. 4. No paper in the hand-writing of the author, or with his name affixed, can be received; and, if the author of any paper shall discover himself to the committee of papers, or to any member thereof, such paper will be excluded from all competition for the medal. 5. The prize essay will be read before the Society, at the meeting preceding the anniversary meeting of the Society, in March 1824. 6. The prize medal will be presented to the successful candidate, or his substitute, at the anniversary meeting of the Society. 7. All the dissertations, the successful one excepted, will, if desired, be returned, with the sealed packets unopened.

One dissertation, only, on the subject "Dropsy," proposed by the Society for the Fothergillian gold medal, to be adjudged in March 1823, having been sent in, the Society, thinking it probable that, in consequence of the recent establishment of the prize, it had not been sufficiently made known to the medical faculty, have deferred the adjudication of the prize for the best dissertation on "Dropsy" to another year.

15. *Provincial Monument to Dr. Jenner.*—At a numerous meeting of medical gentlemen, held at the King's-Head Inn, Gloucester, on the 22d of February, 1823, for the purpose of taking into consideration the best means of testifying their respect for the memory of their lamented countryman, Dr. JENNER, (Dr. BARON in the chair,) it was resolved unanimously, that a subscription should be entered into, for the purpose of erecting a monument to his memory; and the following bankers have been appointed to receive the subscriptions (two guineas each):—Sir James Esdaile and Co., Messrs. Hammond, and Messrs. Ladbroke and Co. London; and Ramsay and Co. Edinburgh.

---

OBITUARY.—Died, on the 22d instant, Dr. HAIGHTON, Lecturer on Midwifery in the Borough.

---

### MONTHLY CATALOGUE OF MEDICAL BOOKS.

### METEOROLOGICAL JOURNAL.

By Messrs. WILLIAM HARRIS and Co. 50, Holborn, London.

*From February 20, to March 19, 1823.*

| | MOON. | Rain gauge | THERM. | | | BAROM. | | DE LUC'S HYG. | | WIND. | | ATMOSPHERIC VARIATION. | | |
|---|---|---|---|---|---|---|---|---|---|---|---|---|---|---|
| | | | 9 A.M. | MAX. | MIN. | 9 A.M. | 10P.M. | 9 A.M. | 10P.M. | 9 A.M. | 10P.M. | 9 A.M. | 2 P.M. | 10P.M. |
| Feb. | | | | | | | | | | | | | | |
| 20 | | | 38 | 43 | 36 | 29.67 | 29.80 | 71 | 72 | W | WSW | Fine | | |
| 21 | | .21 | 39 | 48 | 33 | 29.53 | 29.36 | 85 | 80 | WSW | WSW | Rain | | Fine |
| 22 | | | 36 | 42 | 36 | 29.50 | 29.42 | 90 | 75 | W var. | WSW | Rain | | Fine |
| 23 | | | 39 | 46 | 39 | 29.59 | 29.21 | 96 | 78 | W var. | WSW | Rain | Stormy | Fine |
| 24 | | .77 | 43 | 50 | 38 | 29.53 | 29.61 | 68 | 79 | NWva | W var. | Fine | | V. fine |
| 25 | ○ | | 41 | 49 | 34 | 29.45 | 29.98 | 88 | 84 | SE | W str. | Cloudy | Rain | R. stW |
| 26 | | | 37 | 45 | 32 | 28.80 | 28.92 | 68 | 79 | SW | WSW | Fine | | Ha.Cl. |
| 27 | | | 36 | 48 | 34 | 29.05 | 29.13 | 77 | 72 | NNW | NW | Fine | | |
| 28 | | .06 | 37 | 43 | 33 | 29.31 | 29.51 | 71 | 75 | WNW | WNW | Fair | Fine | |
| Mar. | | | | | | | | | | | | | | |
| 1 | | | 35 | 49 | 34 | 29.74 | 29.88 | 77 | 84 | NNW | NWva | Fine | | Cloud. |
| 2 | | | 36 | 50 | 44 | 29.86 | 29.66 | 75 | 80 | SW | SWvar | Fine | | Sho'ry |
| 3 | | | 46 | 50 | 40 | 29.50 | 29.29 | 84 | 74 | SW | WSW | Cloudy | Sho'ry | Cloud. |
| 4 | ☽ | | 42 | 46 | 37 | 29.19 | 29.15 | 68 | 75 | W var. | W hur. | Fine | | Fair |
| 5 | | | 39 | 45 | 32 | 29.25 | 29.43 | 70 | 77 | WNW | WNW | Fine | Sho'ry | |
| 6 | | .10 | 34 | 39 | 31 | 29.60 | 29.64 | 77 | 67 | NNW | NW | Fair | Fine | |
| 7 | | | 33 | 33 | 32 | 29.30 | 28.84 | 73 | 90 | SSE | SE | Cloudy | Snow | Sho'ry |
| 8 | | | 34 | 40 | 32 | 28.92 | 29.00 | 84 | 80 | WSW | NEvar | Fair | Fine | Fair |
| 9 | | | 36 | 40 | 31 | 29.10 | 29.44 | 79 | 70 | N var. | NNW | Fair | Fine | |
| 10 | | .20 | 33 | 39 | 36 | 29.58 | 29.37 | 77 | 80 | W | SSE | Cloudy | Fair | Rain |
| 11 | | | 39 | 48 | 35 | 29.54 | 29.69 | 74 | 75 | WSW | W | Fine | | |
| 12 | ● | | 38 | 50 | 37 | 29.91 | 30.02 | 79 | 70 | WNW | NW | Fine | | |
| 13 | | | 40 | 50 | 45 | 30.07 | 30.04 | 70 | 75 | W | SSW | Fine | Fair | Misty |
| 14 | | | 47 | 52 | 39 | 30.04 | 30.13 | 83 | 83 | SW | NNE | Foggy | Rain | Sho'ry |
| 15 | | | 43 | 50 | 35 | 30.20 | 30.24 | 83 | 85 | NNE | ENE | Showery | Fair | Cloud. |
| 16 | | | 37 | 49 | 37 | 30.24 | 30.08 | 87 | 84 | NNE | SW | Cloudy | Fine | Foggy |
| 17 | | | 39 | 48 | 40 | 29.95 | 29.87 | 87 | 81 | NNW | WNW | Showery | Cloud. | |
| 18 | | | 43 | 50 | 32 | 29.60 | 29.45 | 79 | 83 | SW | NNW | Showery | Rain | Fine |
| 19 | ☾ | .37 | 32 | 40 | 31 | 29.50 | 29.77 | 76 | 74 | N | NNW | Sn. & R. | Fair | Fine |

The quantity of rain during the month of February, was 2 inches and 87.100ths.

